# Ruptured Brain Arteriovenous Malformation Coexisting With Reversible Cerebral Vasoconstriction Syndrome: A Case Report

**DOI:** 10.7759/cureus.100423

**Published:** 2025-12-30

**Authors:** Kessarin Panichpisal, Melanie B Fukui, Amin B Kassam, Mohammad Anadani, Rehan Sajjad

**Affiliations:** 1 Neurology, Intent Medical Group, Endeavor Health Advanced Neurosciences Institute, Northwest Community Hospital, Arlington Heights, USA; 2 Radiology, Intent Medical Group, Endeavor Health Advanced Neurosciences Institute, Northwest Community Hospital, Arlington Heights, USA; 3 Neurological Surgery, Intent Medical Group, Endeavor Health Advanced Neurosciences Institute, Northwest Community Hospital, Arlington Heights, USA

**Keywords:** acute subdural hematoma, brain arteriovenous malformation, convexity subarachnoid hemorrhage, intracranial hemorrhage, reversible cerebral vasoconstriction syndrome

## Abstract

Reversible cerebral vasoconstriction syndrome (RCVS) is known to coexist with other vascular lesions; however, the coexistence of a ruptured brain arteriovenous malformation (AVM) and RCVS has not been previously reported. We describe the case of a 48-year-old woman who presented with an acute thunderclap headache followed by left-sided weakness. A noncontrast head CT revealed an acute right frontal intracerebral hemorrhage, extensive subarachnoid hemorrhage, and a right posterior fossa subdural hematoma, a pattern atypical for isolated RCVS. Cerebral angiography demonstrated multifocal vasoconstriction consistent with RCVS. The patient underwent hematoma evacuation, during which a ruptured brain AVM was identified and resected, with histopathologic confirmation. Postoperative cerebral angiography demonstrated no residual AVM. The patient experienced substantial neurological recovery following rehabilitation, with only mild residual left-sided weakness at follow-up. This case underscores the importance of maintaining a high index of suspicion for coexisting vascular lesions in patients with presumed RCVS who present with atypical hemorrhagic patterns.

## Introduction

Reversible cerebral vasoconstriction syndrome (RCVS) is one of the most common causes of acute thunderclap headache, aside from aneurysmal subarachnoid hemorrhage (SAH). It is characterized by reversible, segmental narrowing of the cerebral arteries and most frequently affects women in their fourth decade of life [1]. Although RCVS can occur spontaneously, the majority of patients have an identifiable trigger or associated condition, including sexual activity, Valsalva maneuver, a history of migraine, or exposure to sympathomimetic or serotonergic agents [1,2].

RCVS may lead to a range of complications, including intracranial hemorrhage, ischemic stroke, reversible cerebral edema, and seizures [2]. In addition, a high prevalence of coexisting vascular lesions has been reported in patients with RCVS, including cervical artery dissection (CAD), unruptured aneurysms, fibromuscular dysplasia (FMD), developmental venous anomalies (DVAs), and cavernous malformations (CMs) [3]. To our knowledge, we present the first reported case of a ruptured brain arteriovenous malformation (AVM) coexisting with RCVS.

## Case presentation

A 48-year-old woman with a history of bipolar disorder, anxiety, schizoaffective disorder, and migraine presented with the sudden onset of the worst headache of her life during orgasm, accompanied by left-hand numbness. She denied any preceding headache, neurological symptoms, or sentinel events in the days leading up to presentation. The headache resembled her prior migraines, which were occasionally associated with transient paresthesias. She took sumatriptan 100 mg, a known trigger for RCVS, without relief, repeated the dose two hours later, and went to bed. Approximately three hours later, she awoke with new left-sided weakness and was transported to the hospital by emergency medical services. Her home medications included sumatriptan and topiramate for migraine. She was also taking desvenlafaxine and bupropion, which have been reported as potential triggers for RCVS, as well as cariprazine for psychiatric indications [1]. She reported tobacco use and occasional alcohol consumption but denied illicit drug use. There had been no recent medication changes.

**Figure 1:**
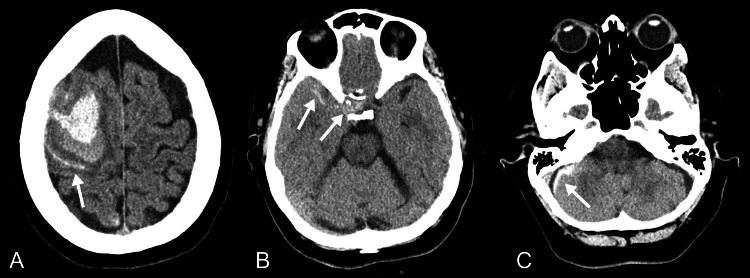
Initial unenhanced head CT images. (A) Axial images demonstrate a right frontal intraparenchymal hematoma with adjacent convexity SAH (arrow). (B) Additional SAH is present within the right Sylvian fissure and suprasellar cistern (arrows). (C) A small right posterior fossa subdural hematoma is also noted (arrow). CT = Computed Tomography; SAH = Subarachnoid Hemorrhage.

On arrival, her blood pressure was 143/102 mm Hg, and the neurological examination revealed dense left hemiplegia. A noncontrast head CT demonstrated an acute right posterior frontal intracerebral hemorrhage (ICH) measuring approximately 3.9 × 3.1 × 3.0 cm, with adjacent subarachnoid extension into the right sylvian fissure and suprasellar cistern, a small right parietal cortical SAH, and an acute right posterior fossa subdural hematoma (SDH) (Figure 1). CT angiography revealed irregularities of the distal anterior cerebral arteries and vasospasm involving the distal posterior cerebral arteries.

**Figure 2:**
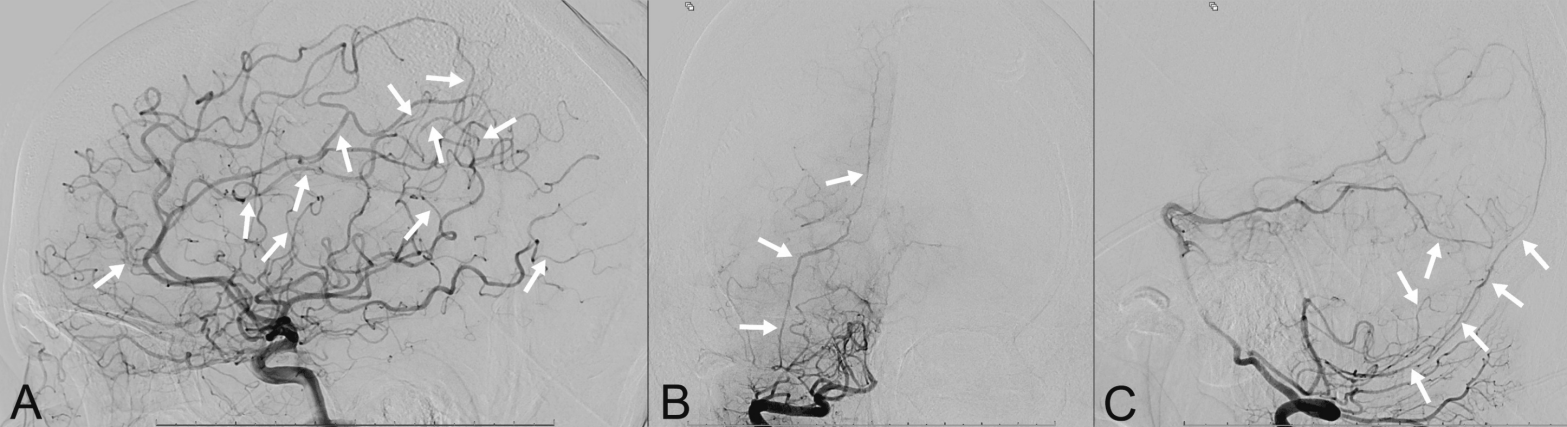
Cerebral angiography images. (A) Right internal carotid artery injection demonstrates multifocal vasoconstriction of the distal anterior cerebral arteries and right middle cerebral artery branches (arrows). (B, C) Right vertebral artery injection shows mild distal vasospasm of the right posterior cerebral and posterior inferior cerebellar arteries, with long-segment alternating vasoconstriction and vasodilation of the right posterior meningeal artery (arrows).

Cerebral angiography (Figure 2) demonstrated multifocal distal anterior cerebral artery stenosis, mild distal middle cerebral artery narrowing (most apparent on the left), and posterior cerebral artery narrowing, and long-segment alternating constriction and dilatation of the right posterior meningeal artery, findings consistent with RCVS [4]. Importantly, no AVM nidus was visualized on this preoperative study despite the presence of a large intracerebral hemorrhage. A 1.5-mm left paraophthalmic internal carotid artery aneurysm was also identified. Based on the clinical presentation and angiographic findings, the initial diagnostic impression favored hemorrhage secondary to RCVS.

She underwent a craniotomy with hematoma evacuation, during which a small brain AVM was unexpectedly discovered and resected. Histopathologic examination demonstrated that the majority of the specimen consisted of extensive blood clots and hematomas. In several foci, vessels of varying calibers were identified, including abnormal small- to medium-sized thick-walled muscular veins, venules, and hybrid vessels, findings compatible with a vascular malformation. Intervening gliotic brain parenchyma and features of chronic hemorrhage were not identified due to the absence of surrounding brain tissue in the specimen; nevertheless, the abundant associated blood clot provided unequivocal evidence of acute hemorrhage. Postoperative angiography demonstrated no residual AVM. Because the AVM was angiographically occult preoperatively and discovered incidentally at surgery, a Spetzler-Martin grade could not be reliably assigned. Her headache was managed with oxycodone, celecoxib, gabapentin, verapamil, and topiramate, and both triptan and antipsychotic medications were discontinued.

On hospital day five, the patient developed recurrent thunderclap headaches. Repeat noncontrast head CT demonstrated no new hemorrhage, while CT angiography demonstrated interval vasoconstriction involving more proximal intracranial vessels. Headache management was escalated with the initiation of intravenous magnesium infusion, resulting in symptomatic improvement, and she was subsequently transferred to an acute inpatient rehabilitation unit.

Approximately one week later, while in the rehabilitation unit, she reported bilateral blurry vision. Ophthalmologic evaluation revealed acute papilledema, prompting transfer back to the neurological floor for further evaluation. CT venography was negative for venous sinus thrombosis or stenosis, and repeat head CT demonstrated mild enlargement of the third ventricle. She subsequently underwent left laparoscopic-assisted ventriculoperitoneal shunt placement, with improvement in visual symptoms. She returned to the rehabilitation unit and completed approximately one month of inpatient rehabilitation before being discharged home with near-normal strength in the left leg and moderate residual weakness in the left arm. Approximately one week after discharge, she developed left frontal incisional drainage, necessitating removal of the ventriculoperitoneal shunt. Follow-up ophthalmologic evaluation demonstrated near-complete resolution of papilledema, and she was treated with acetazolamide. Subsequent examinations confirmed complete resolution of papilledema. A follow-up cerebral angiogram was planned at three months.

## Discussion

This case underscores the importance of recognizing uncommon coexisting vascular lesions, such as a ruptured brain AVM, in patients with RCVS. The patient presented with a classic thunderclap headache and had multiple established RCVS triggers, including triptan use. Angiography demonstrated multifocal, segmental vasoconstriction involving distal intracranial vessels, a pattern characteristic of true RCVS and distinct from secondary vasospasm related to intracranial hemorrhage, which is typically localized to vessels adjacent to the site of bleeding. Although triptan use and intracranial hemorrhage may contribute to cerebral vasoconstriction, the diffuse, multifocal distribution and involvement of vascular territories beyond the hemorrhage support a diagnosis of true RCVS rather than isolated drug- or hemorrhage-induced vasospasm [4].

Hemorrhagic complications have been reported in approximately 40% of RCVS cases, with female sex and older age identified as independent risk factors [2,5]. The hemorrhagic spectrum includes SAH, ICH, and, less commonly, SDH or intraventricular hemorrhage [2,5-7]. SAH in RCVS typically manifests as a focal cortical hemorrhage limited to one or more sulci, either unilaterally or bilaterally. In contrast, diffuse SAH extending into the perimesencephalic cisterns or sylvian fissure is distinctly uncommon. SDH is rare and generally occurs in association with other hemorrhagic patterns. Notably, up to one-third of patients with RCVS may exhibit mixed hemorrhagic types [2,5,6].

In our patient, the hemorrhage was more extensive than is typically observed in RCVS, involving not only the cortical surface but also the cisternal and sylvian compartments. Although such a hemorrhagic distribution could raise concern for an aneurysmal source, comprehensive catheter angiography demonstrated no intracranial aneurysm at or near the site of bleeding. Instead, the location and extent of hemorrhage, including a large frontal ICH with associated cisternal SAH, were anatomically concordant with the ruptured frontal AVM, supporting a contributory role of the AVM in the observed hemorrhagic pattern.

In contrast, the right posterior fossa SDH was more likely related to RCVS itself, given the angiographic findings of alternating vasoconstriction and dilatation involving the posterior meningeal artery. This atypical and mixed hemorrhagic distribution prompted concern for an additional underlying lesion and ultimately led to the identification of a ruptured AVM. Such findings reinforce the importance of maintaining a high index of suspicion for coexisting vascular malformations in patients with presumed RCVS when hemorrhagic patterns deviate from the classic focal cortical presentation. Importantly, the AVM in this case might have remained undetected without hematoma evacuation, underscoring the value of careful surgical inspection of the hematoma bed when performed, as well as repeat catheter angiography beyond noninvasive imaging alone in cases with atypical clinical or radiographic findings, since small or obscured vascular lesions may otherwise be missed [8].

Crucially, the preoperative catheter angiogram (Figure 2) did not demonstrate an AVM nidus, despite the presence of a large ICH. The angiographically occult nature of the AVM, despite intraoperative visualization and histopathological confirmation, represents a key and perplexing finding in this case. Several mechanisms may account for this discrepancy. A very small nidus may have been obscured by the surrounding hematoma and mass effect, rendering it angiographically silent [9]. Alternatively, the lesion may represent a micro-AVM that underwent partial or transient thrombosis following rupture, resulting in temporary angiographic occultation, consistent with a cryptic AVM [10]. Subsequent hemodynamic instability may have contributed to re-rupture of the residual fragile vascular channels. In this context, coexisting RCVS may have further obscured the lesion by reducing flow through a diminutive feeder or nidus and promoting thrombosis. Collectively, these mechanisms highlight the limitations of angiography in select hemorrhagic presentations and underscore the diagnostic complexity of small or angiographically occult AVMs.

To our knowledge, this represents the first reported case in the English-language literature of a ruptured brain AVM coexisting with RCVS. Prior studies have described a relatively high prevalence of other coexisting neurovascular lesions in RCVS, including CAD, unruptured aneurysms, CMs, FMD, and DVAs. These associated abnormalities are typically asymptomatic, except when an arterial dissection results in ischemia. Their presence suggests that patients with RCVS may harbor subtle structural susceptibilities that predispose them to both vasoconstriction and vascular wall fragility [3,11].

The exact pathophysiology of RCVS remains uncertain. Proposed mechanisms include dysregulation of cerebrovascular tone, blood-brain barrier disruption, endothelial dysfunction, oxidative stress, sympathetic overactivity, and altered trigeminovascular nociception [1,12]. RCVS is thought to follow a centripetal progression, with initial involvement of distal small arteries that undergo segmental dilatation and abrupt stretching, predisposing to vessel rupture or reperfusion injury. As the process advances, more proximal arteries develop segmental vasoconstriction, which may result in watershed infarction [6,7].

The hemodynamic oscillations characteristic of RCVS, alternating vasoconstriction and reactive vasodilation, may induce abrupt fluctuations in cerebral perfusion pressure. Such cycles can transiently increase wall shear stress on structurally abnormal AVM vessels, particularly in the setting of endothelial dysfunction and impaired autoregulation, thereby predisposing them to rupture. In patients with AVM, abnormal vessel wall architecture confers inherent vulnerability to hemorrhage, and sudden hemodynamic shifts during the vasodilatory phase of RCVS may further amplify stress on the nidus and precipitate rupture [6,13].

Management of coexisting RCVS and ruptured AVM requires a multidisciplinary approach. Initial priorities include hemodynamic stabilization, avoidance of vasoactive triggers, and symptomatic management of RCVS. Calcium-channel blockers are commonly administered in clinical practice for headache control in RCVS, although they do not directly reverse vasoconstriction [14]. In this patient, complete surgical resection of the AVM was achieved and confirmed by a negative postoperative catheter angiogram. Should follow-up angiography demonstrate a residual nidus, radiosurgery with or without adjunctive embolization could be considered. Coordinated care among neurologists, neurointerventionalists, and neurosurgeons is critical in such complex presentations.

Previous literature has primarily described the coexistence of RCVS with aneurysmal rupture or arterial dissection [11,15]. The presence of a ruptured AVM broadens the recognized spectrum and suggests that overlapping hemodynamic and endothelial factors may contribute to both vasoconstriction and vascular wall fragility. Further investigation is warranted to clarify the mechanisms linking RCVS with structural vascular abnormalities.

## Conclusions

In summary, this case expands the clinical spectrum of hemorrhagic RCVS and underscores the importance of considering coexisting vascular malformations when hemorrhagic patterns are atypical. In such complex presentations, maintaining a low threshold for repeat or delayed catheter angiography and, when clinically appropriate, careful surgical inspection is essential, as small or obscured vascular lesions may be missed on initial noninvasive imaging. Early recognition and coordinated multidisciplinary management are critical to optimizing clinical outcomes.
